# Delayed-Start Study Design for Balloon Dilation of the Eustachian Tube: Alternative for a Randomized Controlled Trial

**DOI:** 10.3389/fsurg.2017.00010

**Published:** 2017-02-20

**Authors:** Shari Van Roeyen, Paul Van de Heyning, Vincent Van Rompaey

**Affiliations:** ^1^Faculty of Medicine and Health Sciences, University of Antwerp, Antwerp, Belgium; ^2^Department of Otorhinolaryngology and Head & Neck Surgery, Antwerp University Hospital, Edegem, Belgium

**Keywords:** Eustachian tube, questionnaires, diagnosis, tests, research design

## Introduction

The Eustachian tube (ET) is a narrow canal that courses from the middle ear cavity to the nasopharynx, which opens to provide ventilation to the middle ear and to equalize middle ear and ambient pressures (1). In this article, we refer by “Eustachian tube dysfunction” only to obstructive ET dysfunction, i.e., failure to open and ventilate the middle ear, as opposed to patulous ET dysfunction, in which there is failure of ET closure. ET dysfunction is a common medical problem with an estimated incidence of 1% in the adult population and produces symptoms of aural fullness, otalgia, tinnitus, and hearing loss, often exacerbated or precipitated by atmospheric pressure changes (2, 3). Upper airway inflammation (including allergic rhinitis and chronic rhinosinusitis) commonly precipitate (episodes of) obstructive ET dysfunction (1). Long-lasting ET dysfunction may also result in middle ear conditions such as serous otitis media, tympanic membrane (TM) retractions, and cholesteatoma (4).

## Lack of High-Quality Evidence for Balloon Dilation of the ET (BDET)

Apart from potential drug-based therapies mainly focusing on comprehensive treatment of chronic rhinosinusitis, BDET has been suggested as a safe and feasible treatment option in cases refractory to medical treatment (5). During this procedure under general anesthesia, a balloon is introduced transnasally in the cartilaginous portion of the ET and inflated to a 20-mm length and a 3-mm width to a pressure of 10 bar for 2 min. However, the experience on BDET—results only reported in case series—has not reached an adequate level of evidence to support widespread clinical use (6). Ideally, a randomized double-blinded placebo-controlled trial (RCT) should be performed to achieve the highest level of evidence. Although an RCT is the “gold standard” to assess the efficacy of any intervention, we believe it is ethically not justifiable to perform “sham surgery,” in which the patient would be anesthetized but no surgical act performed. Therefore, we suggest an alternative study design to investigate the effect of BDET and achieve a comparable level of evidence: the *delayed-start study design*.

## Delayed-Start Study Design

**Figure 1:**
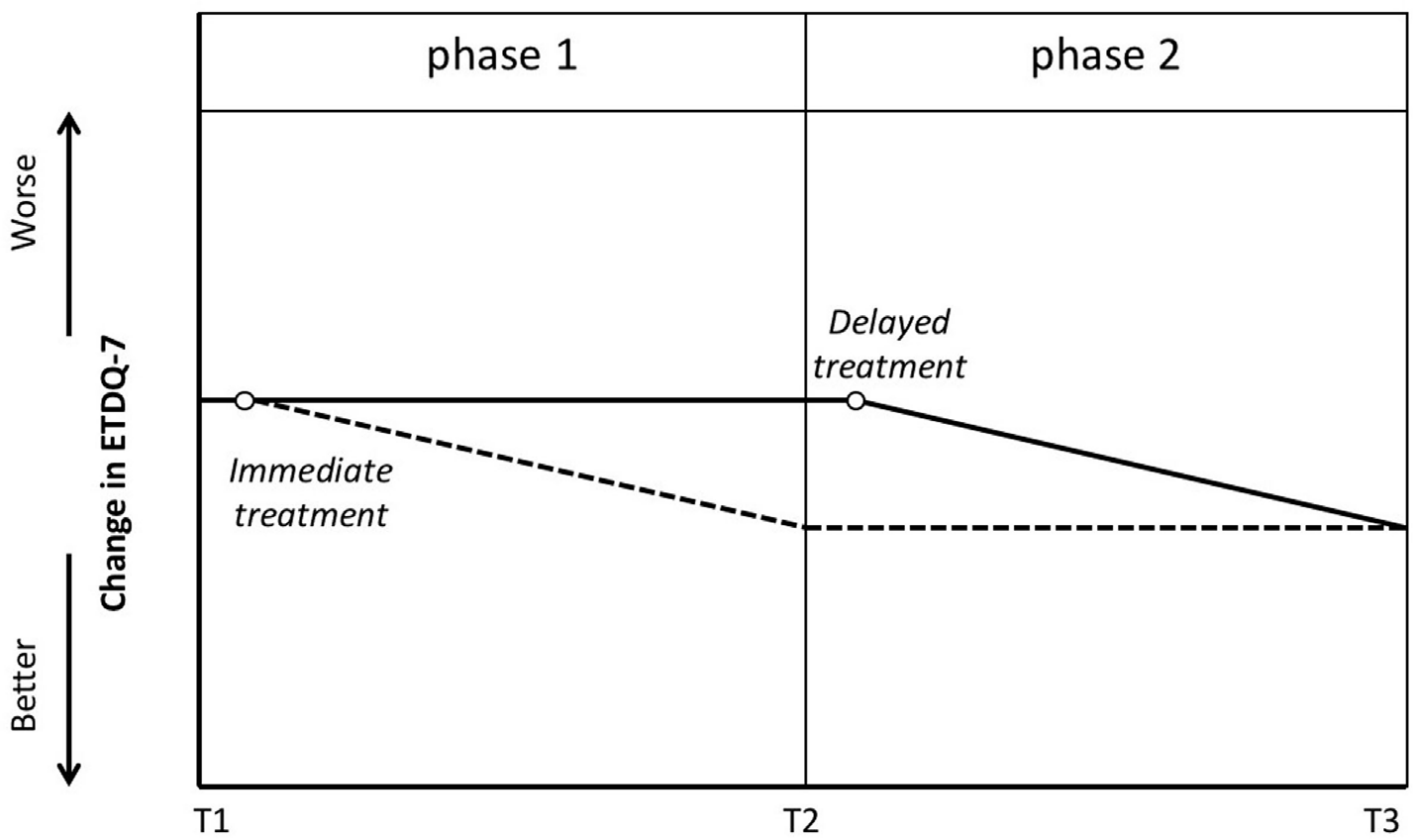
**Delayed-start study design**. Blinded assessment at T1 with subsequent randomization. One group will receive treatment (empty circle) shortly after T1 and will be evaluated again at T2 and T3. The dotted line suggests the potential improvement in Eustachian Tube Dysfunction Questionnaire (ETDQ-7) in this group. The other group will be put on a waiting list and reevaluated at T2. The solid line suggests that these patients have stable symptoms at T2. Shortly after T2 they will receive treatment (empty circle). The latter group will be evaluated again at T3.

The prospective delayed-start study design includes blinded assessment moments at baseline, after phase 1, and after phase 2 (7). At baseline, patients would have to be evaluated and will be randomly assigned to either the group that will receive immediate treatment or a group with a waiting time for the treatment, generally 12 weeks (Figure 1). While one group will receive (immediate) treatment at the beginning of phase 1, another group will be assessed at the end of phase 1 without receiving any treatment. The latter group will then receive (late) treatment, while the first group will be reassessed twice (at the end of phase 1 and phase 2). Both groups will be reassessed after phase 2. Ideally at the end of phase 1, the assessment would demonstrate statistical significance between the treated group and the group that was not treated yet. At the end of phase 2 (when all patients are treated), this statistically significant difference would disappear. Intermediate measures could include repeated scoring by means of the Eustachian Tube Dysfunction Questionnaire (ETDQ-7).

## Minimal ET Outcome Measures

There are a wide range of ET function tests including impedance audiometry (8), otoscopy (9), endoscopic findings (10), Eustachian Tube Score (11), tympanometry (4, 12), Valsalva maneuver (9, 11, 12), and pure-tone audiometry, but none can be considered as a gold standard. The optimal assessment tool for ET dysfunction lies probably in a combination of objective clinical tests and patient-reported outcome measures (13, 14). At each time point, an identical set of outcome measures should be used. We would prefer to use a combination including tympanometry, tubomanometry, and the ability to perform Valsalva maneuver during otoscopy. The 7-item ETDQ-7 and the Eustachian Tube Score 7 (ETS-7) can be used as patient-reported outcome measures. However, none of the suggested diagnostic tools are uniformly able to determine the adequacy of tubal function.

### Tympanometry

Tympanometry is one of the few tests for ET dysfunction in regular clinical use. Though the technique is fast and robust, it is limited by middle ear pressure that can vary considerably over the course of a few hours. Consequently, a single measure of middle ear pressure might not provide enough information on ET opening (15).

### Tubomanometry

The use of tubomanometry in clinical practice is still limited. Initial research suggested that this test can detect ET dysfunction with a sensitivity of 49% and specificity of 93% for opening threshold measurements, and a sensitivity of 87% and specificity of 67% for latency measurements (13). Its main limitations are that it is time-consuming and has a significant inter-operator variability (16).

### Otoscopy

During otoscopy features like middle ear effusion or TM retraction can be suggestive for ET dysfunction. However, it is an insensitive measure of physiological performance of the ET and should rather be considered as a complication of ET dysfunction. As an alternative, patients with an aerated middle ear can perform a Valsalva maneuver while the TM is being visualized. When the patient indicates a change in aural pressure or the TM moves during the maneuver, it is suggestive for patency of the ET. However, up to one-third of healthy individuals fail this test (17).

### The 7-Item ETDQ-7

The ETDQ-7 is a disease-specific instrument for the assessment of symptoms related to obstructive ET dysfunction. It is the only symptom score of ET dysfunction to have undergone validation studies (18–20). The ETDQ-7 cannot discriminate between patients with obstructive and patulous ET dysfunction. However, we believe it is a precise measure of symptoms in patients with obstructive ET dysfunction (19). A recent study by our group showed that this questionnaire is responsive to change in patients with baro-challenge-induced ET dysfunction who have undergone BDET (20).

### The ETS-7

The ETS-7 has been reported as a promising diagnostic tool for obstructive ET dysfunction (21). An advantage of the ETS-7 is the inclusion of objective and subjective elements including tubomanometry, tympanometry, and objective Valsalva.

## Conclusion and Future Directions

Clinical research in patients with ET disorders still suffers from the lack of high-quality evidence. The health technology assessment by Llewellyn et al. (6) identified the variability in inclusion criteria and unclear and variable definitions for ET dysfunction as one of the principal findings. Therefore, definitions and diagnostic criteria were suggested in the 2015 consensus statement by Schilder et al. (14), which provides a significant step toward the design of future (high-quality) trials. In this consensus statement, RCTs were suggested to study change after treatment and to improve the level of evidence. Indeed, ideally an RCT should be performed to achieve the highest level of evidence. Although the surgeon is not blinded to the treatment allocation, blinded investigators can help to establish outcome. The most important consideration, however, is the ethical issue related to “sham surgery,” in which the patient would be anesthetized but no surgical act to be performed, or only one side is treated in bilateral cases (14). For this reason, we suggest an alternative study design to investigate treatment effect and achieve a comparable level of evidence: the *delayed-start study design* (also called the randomized-start design) (7). Although this study design shows great promise and regulatory authorities have shown to support this approach, few clinical researchers are aware of it.

In our opinion, this study design can provide a high-quality alternative to RCTs without potential objections by ethics committees toward randomization of real surgery and sham surgery. The optimal test (i.e., validated for the condition) for studying serous otitis media would be tympanometry, and in case of baro-challenge-induced ETD, tubomanometry and the ETDQ-7 are the evaluation methods.

## Author Contributions

VS, PH, and VR provided substantial contributions to the conception or design of the work; to the acquisition, analysis, and interpretation of data for the work; and to drafting the work or revising it critically for important intellectual content; gave final approval of the version to be published; and agreed to be accountable for all aspects of the work in ensuring that questions related to the accuracy or integrity of any part of the work are appropriately investigated and resolved.

## Conflict of Interest Statement

The authors declare that the research was conducted in the absence of any commercial or financial relationships that could be construed as a potential conflict of interest.
